# Nuclear Fragile X Mental Retardation Protein Is localized to Cajal Bodies

**DOI:** 10.1371/journal.pgen.1003890

**Published:** 2013-10-31

**Authors:** Alain Y. Dury, Rachid El Fatimy, Sandra Tremblay, Timothy M. Rose, Jocelyn Côté, Paul De Koninck, Edouard W. Khandjian

**Affiliations:** 1 Centre de recherche, Institut en santé mentale de Québec, Québec, Québec, Canada; 2 Département de psychiatrie et des neurosciences, Faculté de médecine, Université Laval, Québec, Québec, Canada; 3 Seattle Children's Research Institute, Seattle, Washington, United States of America; 4 Department of Cellular and Molecular Medicine and Center for Neuromuscular Disease, University of Ottawa, Ottawa, Ontario, Canada; 5 Département de Biochimie, Microbiologie et Bio-Informatique, Université Laval, Québec, Québec, Canada; Stanford University School of Medicine, United States of America

## Abstract

Fragile X syndrome is caused by loss of function of a single gene encoding the Fragile X Mental Retardation Protein (FMRP). This RNA-binding protein, widely expressed in mammalian tissues, is particularly abundant in neurons and is a component of messenger ribonucleoprotein (mRNP) complexes present within the translational apparatus. The absence of FMRP in neurons is believed to cause translation dysregulation and defects in mRNA transport essential for local protein synthesis and for synaptic development and maturation. A prevalent model posits that FMRP is a nucleocytoplasmic shuttling protein that transports its mRNA targets from the nucleus to the translation machinery. However, it is not known which of the multiple FMRP isoforms, resulting from the numerous alternatively spliced *FMR1* transcripts variants, would be involved in such a process. Using a new generation of anti-FMRP antibodies and recombinant expression, we show here that the most commonly expressed human FMRP isoforms (ISO1 and 7) do not localize to the nucleus. Instead, specific FMRP isoforms 6 and 12 (ISO6 and 12), containing a novel C-terminal domain, were the only isoforms that localized to the nuclei in cultured human cells. These isoforms localized to specific p80-coilin and SMN positive structures that were identified as Cajal bodies. The Cajal body localization signal was confined to a 17 amino acid stretch in the C-terminus of human ISO6 and is lacking in a mouse Iso6 variant. As FMRP is an RNA-binding protein, its presence in Cajal bodies suggests additional functions in nuclear post-transcriptional RNA metabolism. Supporting this hypothesis, a missense mutation (I304N), known to alter the KH2-mediated RNA binding properties of FMRP, abolishes the localization of human FMRP ISO6 to Cajal bodies. These findings open unexplored avenues in search for new insights into the pathophysiology of Fragile X Syndrome.

## Author Summary

Fragile X syndrome is the most common form of inherited mental retardation affecting approximately 1/7000 females and 1/4000 males worldwide. The syndrome is due to the silencing of a single gene, the *Fragile Mental Retardation 1* (*FMR1*), that codes for a protein called the Fragile X mental retardation protein (FMRP). This protein, highly expressed in the brain, controls local protein synthesis essential for neuronal development and maturation. While considerable efforts have been focused on understanding FMRP functions in mental retardation, the pathophysiology of the syndrome is not well understood. Here, we show that in addition to the well-studied roles of FMRP in regulating protein synthesis, a minor species of FMRP different from the major one, is specifically found in structures called Cajal bodies present in the cell nucleus. Our observations suggest that different FMRP species, also called isoforms, might have independent cellular functions. These findings might open new avenues in search for new insights in the pathophysiology of Fragile X Syndrome.

## Introduction

Fragile X syndrome, one of the most frequent human genetic diseases, is caused by the silencing of the *FMR1* gene that codes for a heterogeneous set of Fragile X Mental Retardation protein (FMRP) isoforms [1]–[3]. FMRP, particularly abundant in neurons [4], contains two KH domains and an RGG box, both common characteristics amongst RNA-binding proteins [5] and is localized in the cytoplasm. FMRP is a component of messenger ribonucleoprotein complexes present within the translation apparatus [6]–[8], while in neuronal extensions, it is also found in granules containing mRNA that are transported towards autonomous translation micro-domains present in synapses and in growth cones distant from the soma [9], [10].

The most prevalent concept regarding the absence of FMRP is that it causes translation dysregulation and defects in mRNA transport which are thought to alter local protein synthesis essential for synaptic development and maturation [11]. FMRP has been reported to associate with several hundred mRNAs, as detected by high-throughput sequencing of RNAs isolated by cross-linking immunoprecipitation (HITS-CLIP) [12]. A prevalent model posits that FMRP is a nucleocytoplasmic shuttling protein that transports its mRNA targets out of the nucleus [13]–[15]. Despite the fact that FMRP has been observed in the nucleus [16], the nature and potential role(s) of nuclear localized FMRP remain unknown.

Using a new generation of antibodies against FMRP, we present evidence that the most common FMRP isoforms, which are associated with the translation machinery, are not detected in the nucleus. In contrast, FMRP isoforms 6 and 12 (ISO6 and ISO12) [17] were found to be predominantly nuclear and more specifically associated with Cajal bodies. These observations suggest that the nuclear FMRP isoforms may have functions independent from the major cytoplasmic FMRP isoforms. This, in turn, also suggests that nuclear post-transcriptional RNA metabolism could be implicated in the pathophysiology of Fragile X syndrome.

## Results

### FMRP is associated with Cajal bodies

**Figure 1:**
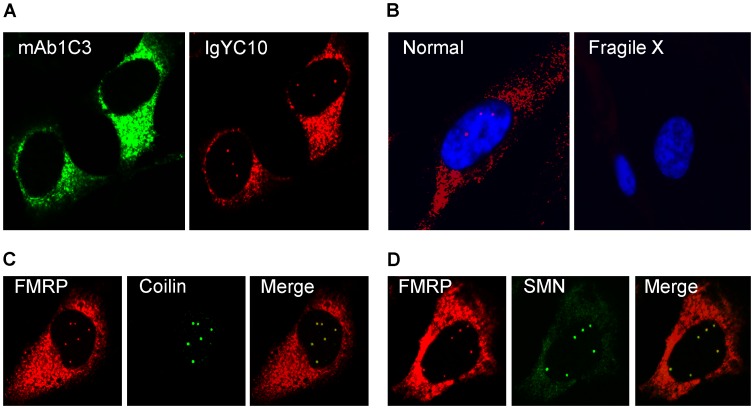
FMRP is present in Cajal bodies. (A) Double immunofluorescence staining of HeLa cells with mAb1C3 (green) and IgY#C10 (red). Note that IgYC10 reveals intranuclear dots that are not seen with mAb1C3. (B) IgYC10 is specific to FMRP in normal human fibroblasts and does not stain any structure in Fragile X fibroblasts derived from a Fragile X donor. Nuclei were stained with DAPI. (C) Colocalization of nuclear FMRP (red) with Coilin (green). (D) Colocalization of nuclear FMRP (red) with SMN (green).

While validating a new generation of antibodies against FMRP raised in chicken [18], we were intrigued by the fact that several batches of IgYs stained, in addition to the classical cytoplasmic distribution, distinct intense dots in the nucleus of HeLa cells. These nuclear dots have not been previously detected using any sera or antibodies raised against FMRP. Double immunostaining of HeLa cells with mAb1C3 [4], a widely used monoclonal antibody against FMRP, and with IgYC10 [18] revealed that both antibodies stained the cytoplasm as expected (Figure 1A). To ascertain that the IgY were specific for FMRP, we affinity-purified the anti-FMRP IgY using recombinant hFMRP. The resulting IgY still reacted with both the nuclear and cytoplamic structures that were eliminated when the immunoreactions were performed in the presence of recombinant hFMRP competitor (data not shown). Although IgYC10 stains strongly the cytoplasm in both human and murine cells in culture, it detects the nuclear structures only in human cells precluding any further analyses in the mouse model. In addition, the detection of FMRP in these nuclear structures by immunofluorescence analyses with the newly developed IgYC10, but not with the classical mAb1C3, strongly suggests that the epitope lying between amino acid 66 to 112 recognized by the latter [4] is not accessible in these structures. We therefore used human fibroblasts to validate whether the nuclear structures were specific to FMRP. Fibroblasts from healthy donors showed the same nuclear and cytoplasmic staining patterns as those observed in HeLa cells, however, the nuclear foci present in >85% of HeLa cells were only detected in <20% of the human fibroblasts. As expected, no FMRP staining was observed in fibroblasts derived from Fragile X patients (Figure 1B), clearly demonstrating the specificity of IgYC10. The round shaped bright nuclear foci, typically two to six in number, reminiscent of Gems in HeLa cells [19], prompted us to investigate whether these FMRP positive structures could correspond to Cajal bodies. These bodies are nuclear structures known to be involved, among others things in histone pre-mRNA transcription and 3′-end processing, as well as in assembly and maturation of RNP complexes, including splicing snRNPs, snoRNPs, scaRNPs and the telomerase RNP [20]–[23]. Double immunostaining with antibodies against Coilin and SMN, marker proteins for Cajal bodies [20]–[23], confirmed that the nuclear FMRP detected with the IgYC10 antibody is indeed associated with these structures (Figure 1C). Similar results were obtained with the human embryonic kidney 293 (HEK293) cells (data not shown).

**Figure 2:**
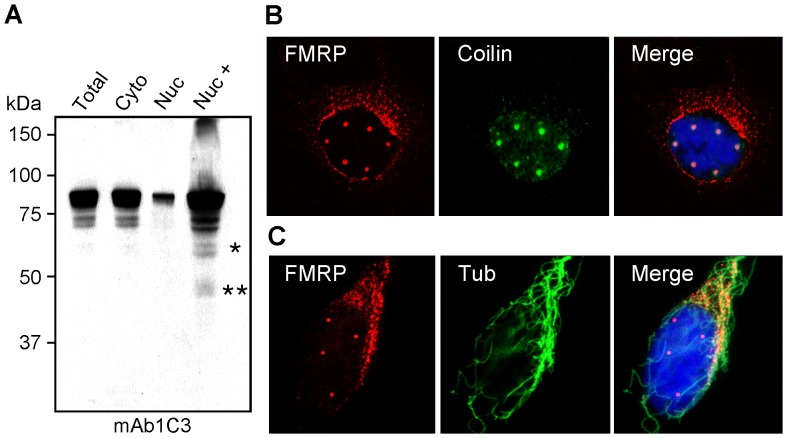
FMRP is present in the isolated nuclear fraction but not in nuclei. (A) Total, cytoplasmic, and nuclear cytoplasmic fractions from HeLa cells were loaded in equal ratios as well as one overloaded nuclear fraction and analyzed by immunoblotting with mAb1C3 to determine the distribution of FMRP. Nuc+ refers to concentrated (20 µg) nuclear protein. (B) Double immunofluorescent localization of FMRP with IgYC10 (red) and Coilin (green) after gentle lysis of the cells *in situ*. Nuclei were counterstained with DAPI. (C) Double immunofluorescent staining of FMRP with IgYC10 (red) and cold-resistant microtubule network revealed with an anti-tubulin antibody (green). Nuclei were counterstained with DAPI. Due to the three dimensional distribution of microtubules, images were taken by conventional epifluorescent microscopy to reveal the microtubule framework.

The observation that FMRP localizes in Cajal bodies raised the question of whether FMRP is only transiently present in the nucleus as part of a shuttling process, or if a specific FMRP sub-population is targeted to the Cajal bodies to remain there. Using standard cell fractionation analyses, it has been estimated that 5 to 10% of total FMRP is recovered within the nuclear fraction (Figure 2A and [15]). However, when we increased the concentration of the nuclear sample (∼20 µg as for the total sample), additional bands of lower molecular weights could be detected (Figure 2A). Either these bands corresponded to new FMRP species, or to degradation products. To our knowledge it has not been proven yet whether this so-called nuclear FMRP is present inside the nucleus or is associated with the nuclear enriched fraction obtained after cell lysis as pointed out by Sittler *et al* [17]. To investigate this question, HeLa cells grown on coverslips were lysed *in situ* in the presence of a buffer containing the non-ionic detergent NP40 to remove most of the cytoplasm, while a cold-resistant cytoskeletal framework containing the cell nucleus [24]–[26] remained attached to the coverslip. After such a treatment, we observed that the FMRP cytoplasmic staining detected with IgYC10 was greatly reduced and was mainly present as perinuclear granular structures outside of the nucleus (Figure 2B) embedded in the cytoskeleton framework, as highlighted using an anti-tubulin antibody (Figure 2C). The same cytoplasmic distribution was also observed using mAb1C3 (Figure S1). On the other hand, FMRP-containing nuclear foci were still detectable following this treatment, strongly arguing that these are indeed nuclear structures.

**Figure 3:**
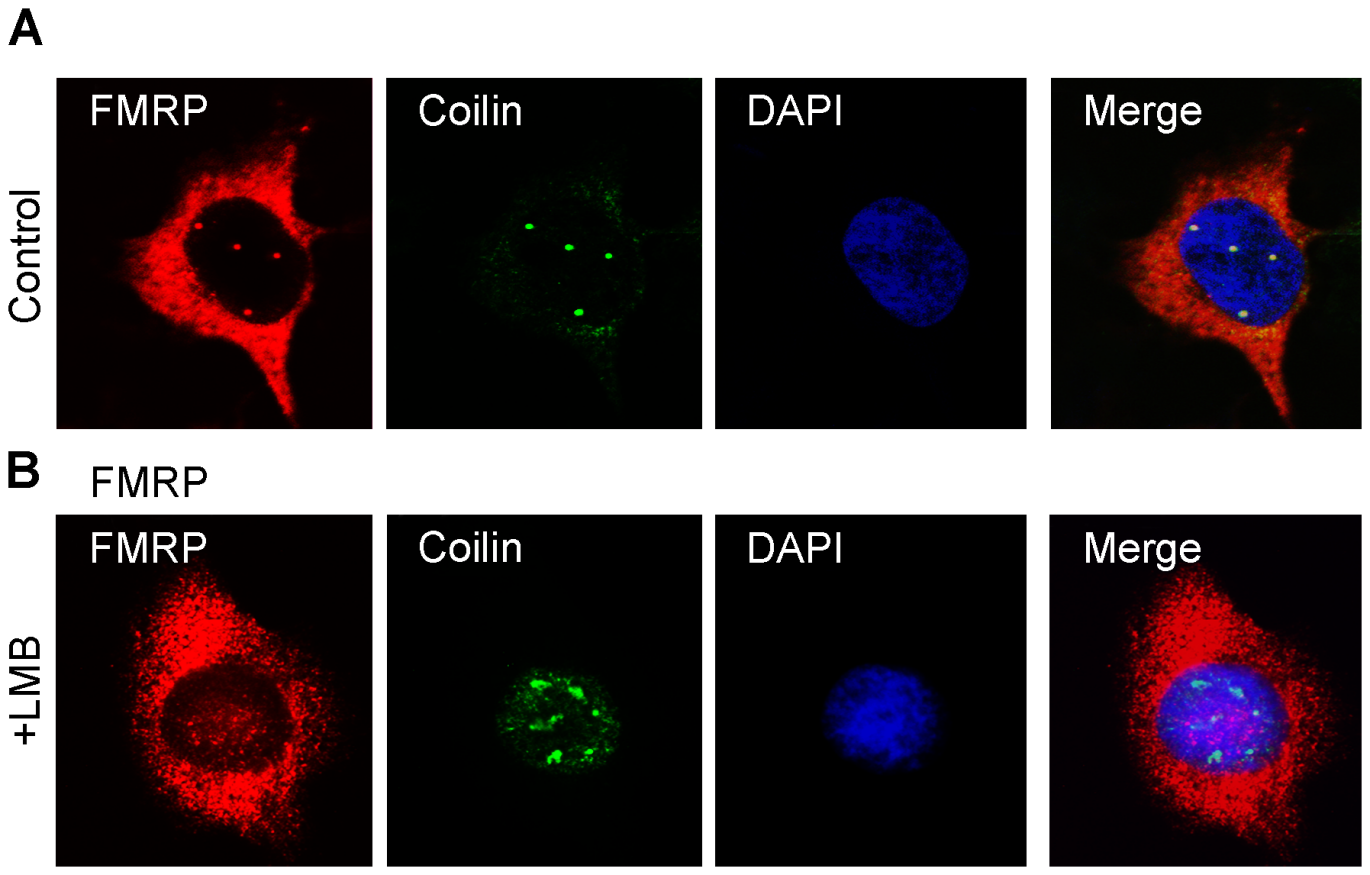
Effects of Leptomycin B on nuclear FMRP localization. Hela cells were maintained in normal conditions (A) or treated with 2 ng/ml LMB for 18 h (B), and then processed for immunofluorescence to localize FMRP (red) and Coilin (green). Nuclei were counterstained with DAPI.

It has been reported that when cells are treated with Leptomycin B (LMB), an inhibitor of the Exportin1/CRM1 pathway of nuclear export of RNA and nuclear proteins containing an NES [27], FMRP accumulates in the nucleus [28]. We therefore treated HeLa cells with 50 ng/ml LMB, as described [28]. However, such a treatment for 20 hours turned to be lethal, indicating strong toxicity of the drug at that concentration. It is noteworthy that the LMB treatment described in [28] was applied 48 hours post-transfection, at a time when cells may contain too much overexpressed FMRP which was shown to cause deleterious effects [29]. Since the turnover of FMRP synthesis and its stability are not known, we hypothesized that a long treatment would be necessary for FMRP to accumulate in the nucleus and prevent its exit. We therefore tested several doses of LMB that could be tolerated for 18 hours, to allow any accumulation of FMRP in the nucleus. We determined that cells could tolerate a treatment of 2 ng/ml (3.7 µM) and remained apparently normal by visual inspection under the microscope. Immunofluorescence with IgYC10 showed that a very slight increase of dispersed endogenous FMRP could be detected in the nucleus of treated cells as compared to untreated cells, which showed distinct staining of Cajal bodies (Figure 3A and B). However this slight increase was likely due to fragmentation and disintegration of Cajal bodies after treatment with LMB and redistribution of its components into nuclear speckles rather than the sequestration of FMRP in the nucleus as claimed [28]. Indeed, double-staining of HeLa cells with IgYC10 and anti-coilin IgG showed that the core protein coilin was redistributed in the nucleoplasm after LMB treatment (Figure 3), as previously documented [30], [31].

Altogether, these results do not provide support for the hypothesis that full length FMRP isoforms (ISO1 or ISO7) shuttle in and out of the nucleus to escort its putative mRNA targets as previously suggested [13]–[15].

### FMRP ISO6 and ISO12 are targeted to Cajal bodies

**Figure 4:**
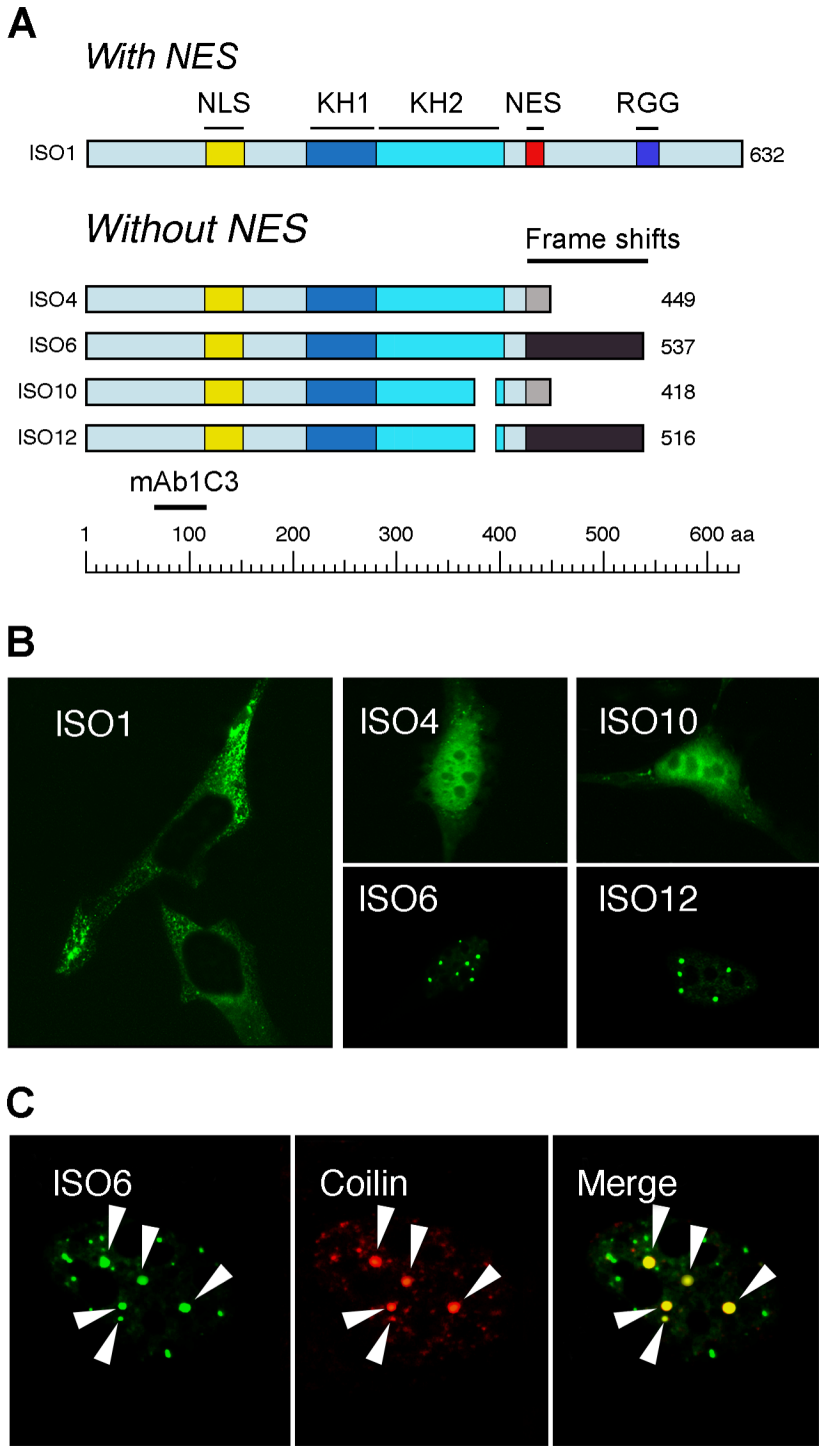
Nuclear ISO6 and ISO12 FMRP are present in Cajal bodies. (A) Structural comparisons between the longest isoform 1 (ISO1) and the nuclear isoforms lacking the NES and RGG domains. Note that all isoforms lacking the NES domain (exon 14) have C-termini different (highlighted in gray) from the main FMRP isoform, due to frame shifts. Note also that mAb1C3 detects all FMRP isoforms. (B) Localization of cytoplasmic and nuclear FMRP isoforms tagged with GFP, after transfection of HeLa cells with the corresponding expression vectors. Note the presence of ISO6 and ISO12 in Cajal bodies, while ISO1 is exclusively localized in the cytoplasm. (C) GFP-ISO6 FMRP colocalizes with Coilin in Cajal bodies (white arrow heads).

Since the *bona fide* full length FMRP detected in nuclear preparations appears to be a perinuclear contaminant (Figure 2), and that GFP-tagged full length FMRP is exclusively cytoplasmic (Figure 4), what would then correspond to the signals detected in Cajal bodies? These unexpected results prompted us to examine whether the new anti-FMRP IgYC10 could recognize nuclear isoforms that have not been detected previously. Indeed, the primary *FMR1* transcripts undergo extensive alternative splicing [32]–[35] leading to numerous potentially different mRNAs as suggested by RT-PCR analyses. Alternative splicing affects the presence of exon 12 and 14 and the choice of acceptor sites in exons 15 and 17 [17]. ISO1 is the longest isoform, while ISO7 lacking exon 12 is the most commonly expressed form in all cells tested. Four short FMRP isoforms (ISO4, 6, 10 and 12) lack the proposed nuclear export signal (NES) and the RGG domains and have C-termini divergent from the major proteins ISO1 and 7 (Figure 4A). The ISO4, 6, 10 and 12 isoforms were shown to have a nuclear localization as detected using transient transfection assays [17].

To determine if one or more of the four nuclear isoforms is present in Cajal bodies, and to test whether a fluorescently tagged version of the nuclear FMRP isoforms would behave similarly to the endogenous protein, we inserted the cDNA coding for each of the four nuclear FMRP isoforms in frame downstream of monomeric GFP cDNA and transfected them in HeLa cells and STEK *fmr1* ^−/−^ KO cells lacking FMRP. Immunoblot analyses of transiently expressed FMRP isoforms in STEK cells are shown in Figure S2. To avoid the formation of stress granules induced by high levels of FMRP [29], and to prevent saturation of the nuclei with FMRP, all analyses were performed within 6 hours post-transfection. As controls, we also followed the fate of GFP-ISO1 FMRP. While the latter isoform was detected exclusively in the cytoplasm (Figure 4B), ISO4, and ISO10 were uniformly distributed in the nucleoplasm while being excluded from nucleoli (Figure 4B). Under the conditions used here, GFP-ISO6 and GFP-ISO12 were predominantly found associated with Cajal bodies as confirmed by their co-localization with Coilin (Figure 4C; not shown for ISO12). Because both ISO6 and 12 localize to Cajal bodies and mimic the structures detected by IgYC10 (see Figure 1), they were analyzed in further studies.

**Figure 5:**
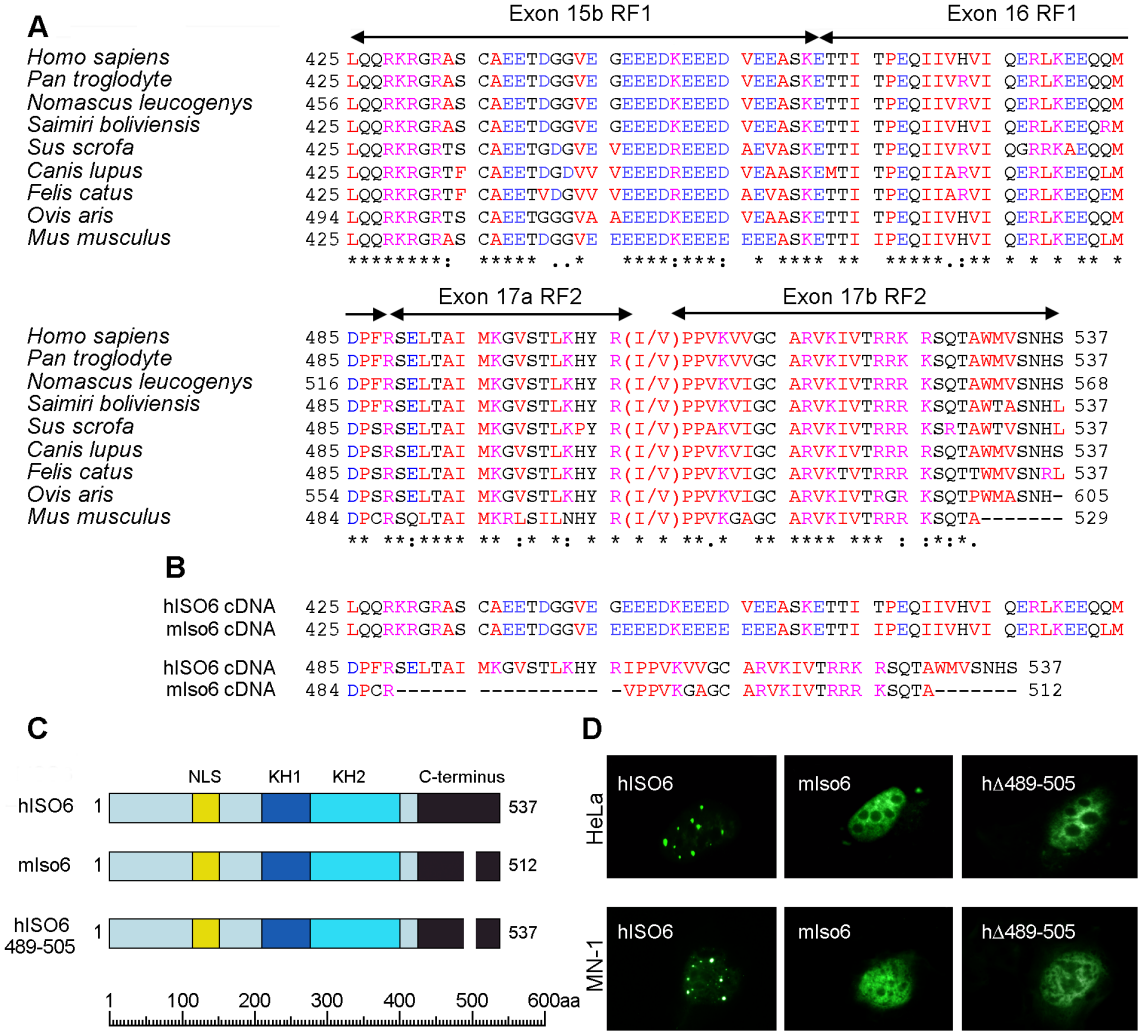
The Cajal body localization signal of human ISO6 is localized to a 17aa C-terminal domain. (A) Evolutionary conserved C-termini of FMRP ISO6. ClustalW multiple sequence alignment of predicted FMRP ISO6 isoforms from different organisms compared to the experimentally determined human ISO6 sequence. Exon positions and numbering are indicated (see Figure S4). GenBank accession numbers : Sus scrofa ref|XP_003360519.1|; Felis catus ref|XP_004000999.1|; Ovis aries ref|XP_004022340.1|; Saimiri boliviensis boliviensis ref|XP_003939137.1|; Canis lupus familiaris ref|XP_003435591.1|; Nomascus leucogenys ref|XP_003271865.1|; Homo sapiens ref|NP_001172004.1| and [17]; Pan troglodytes ref|XP_003317790.1|; Mus Musculus (this study). (B) C-terminal amino acid sequences of hISO6 [17] and a mouse ISO6 variant [35] determined from cloned cDNAs. (C) Schematic representation of GFP hybrids of full-length human ISO6 and the shorter murine ISO6 variant (mIso6) and the GFP-human ISO6 with the 17aa deletion and their localization (D) in human HeLa and murine MN-1 cells after transient transfection.

Previously, sequence analysis of a cDNA encoding human ISO6 [17] revealed that this isoform was generated by alternative splicing of *FMR1* pre-mRNA in which exon 13 was spliced directly to exon 15 at a distal splice acceptor site (Figure S4A), eliminating exon 14 and the sequences encoded in exon 15 from the region between the proximal and distal splice acceptor sites (labeled exon 15a), which are present in ISO1 (reading frame RF3; Figure S4A). Splicing of exon 13 to 15 in ISO6 results in an amino acid sequence derived from reading frame 1 in exon 15b from the distal splice acceptor site (Figure 5A and Figure S4A). This alternate reading frame continues through exon 16 which is spliced to exon 17 (Figure S4B). In human ISO6 cDNA, exon 16 is spliced to exon 17 using a proximal splice acceptor site generating a transcript that encodes an amino sequence in reading frame 2 (RF2; Figure S4C). The alternative splicing detected in the human ISO6 cDNA determined by Sittler et al. [17] is predicted to occur in a variety of other species, as shown in Figure 5A. ISO12 is similar to ISO6 except that it lacks exon 12, which results in shortening of a loop between the β2 and β′ strands within the KH2 domain [36].

A cDNA encoding a variant of human ISO6 (hISO6) has been detected in the mouse [32], [35]. This cDNA is identical in structure to the human ISO6 cDNA except that exon 16 is spliced into exon 17 at a distal splice acceptor site and lacks 17 amino acids encoded by exon17a between the proximal and distal splice acceptors sites (Figure 5B and Figure S5). Since we were unable to detect Fmrp in Cajal bodies in mouse cells such as STEK, 3T3, MN-1, and primary neuron cultures, we hypothesized that the Cajal body localization signal in ISO6 might map to the 17 aa sequence encoded by exon 17a that are missing in the mouse Iso6 variant (Figure 5B and Figure S5). To test this, we first examined the localization of the mIso6 variant lacking the 17aa of exon17a using a construct containing the mIso6 variant cDNA coding sequences (obtained from David Morris, University of Washington, Seattle) fused downstream of GFP (Figure 5C). In both human HeLa and mouse MN-1 cells, the GFP-mIso6 variant gave a general nucleoplasmic localization that was quite distinct from the Cajal body localization seen with the GFP-hISO6 (Figure 5D). Next, we tested the localization of GFP-hISO6 lacking the 17 aa encoded by exon 17a (amino acids 489 to 505) from GFP-hISO6 (Figure 5C). As shown in Figure 5D, the hΔ489-505 ISO6 gave a nucleoplasmic localization in both HeLa and MN-1 mouse cells, similarly to GFP-mIso6 variant.

Bioinformatic analysis of the 17 aa region encoded by exon 17a of hISO6 using the nuclear localization signal (cNLS) Mapper Program [37], identified a cluster of conserved positively charged amino acids (KHxR ; aa 502–505) at the C-terminal end of this region that were predicted to form a bipartite NLS with a second cluster of positively charged amino acids (RRKR ; aa 522–525) encoded within the N-terminal sequence of exon 17b. The disruption of this bipartite NLS in hΔ489-505 ISO6 and in mIso6 may be responsible for the disruption of the Cajal body localization seen with these constructs.

**Figure 6:**
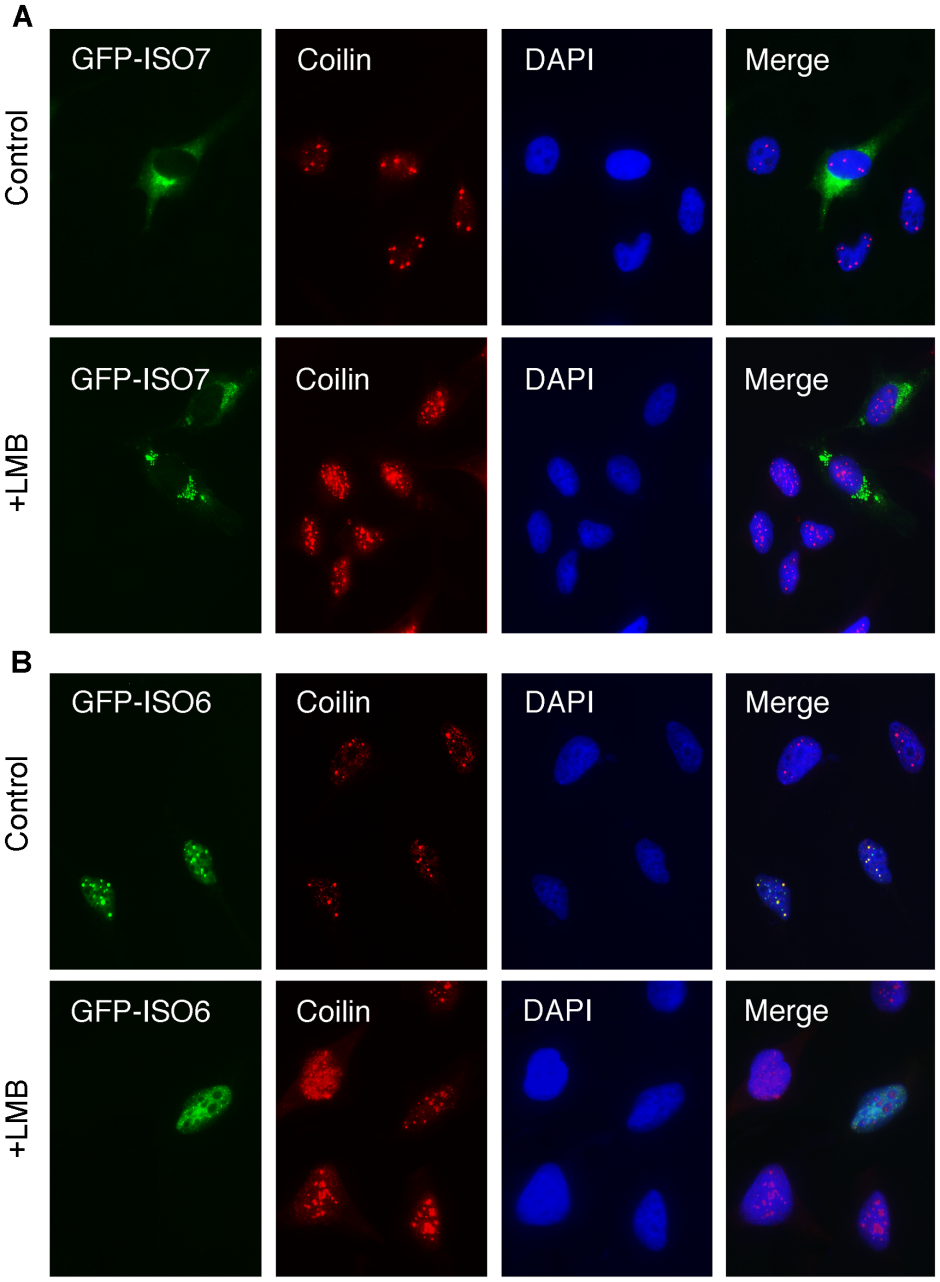
Effects of Leptomycin B on cytoplasmic and nuclear GFP-FMRP localizations. Control and transfected HeLa cells with vectors coding for GFP-ISO7 (A) and GFP-ISO6 (B) were maintained in normal conditions or treated with 2 ng/ml LMB for 20 h, and then processed for immunofluorescence to localize FMRP (green) and Coilin (red). Nuclei were stained with DAPI.

Having shown that endogenous cytoplasmic FMRP does not appear to traffic to the nucleus after treatment with LMB (see above), we confirmed these results using transfection assays. HeLa cells were transfected with vectors encoding GFP-ISO7 and GFP-ISO6. Four hours after transfection, LMB was added to the culture medium at a concentration of 2 ng/ml. After 20 hours of treatment with the drug, cells were processed for immunofluorescence analyses. Control cells with no LMB treatment showed strong GFP-ISO7 cytoplasmic fluorescence and as expected, the presence of FMRP in stress granules due to over-accumulation [29]. The presence of Cajal bodies in the nucleus was assessed using anti-coilin staining, and no difference could be observed between transfected and untransfected cells (Figure 6A). In contrast, LMB treatment induced Cajal bodies to become dispersed, as numerous smaller coilin positive foci were redistributed in the nucleoplasm in both transfected and untransfected cells (Figure 6A). On the other hand, no transfected GFP-ISO7 could be detected in the nucleus after LMB treatment, confirming our previous results with endogenous FMRP (see Figure 3). We next tested the effects of LMB on the nuclear distribution of ISO6. In untreated cells, GFP-ISO6 was associated mainly with Cajal bodies similarly to the endogenous nuclear FMRP detected with the IgYC10 antibody (see Figure 1). After treatment with LMB, GFP-ISO6 was no longer concentrated in Cajal bodies, as it was evenly distributed throughout the nucleoplasm. Also, coilin was redistributed in the nucleoplasm, as smaller coilin positive foci were observed (Figure 6B).

Altogether, these results strongly suggest that the Cajal bodies signals observed for endogenous FMRP with our new antibody likely correspond to ISO6 and/or ISO12 (Figure 3).

### FMRP present in Cajal bodies is cleaved

**Figure 7:**
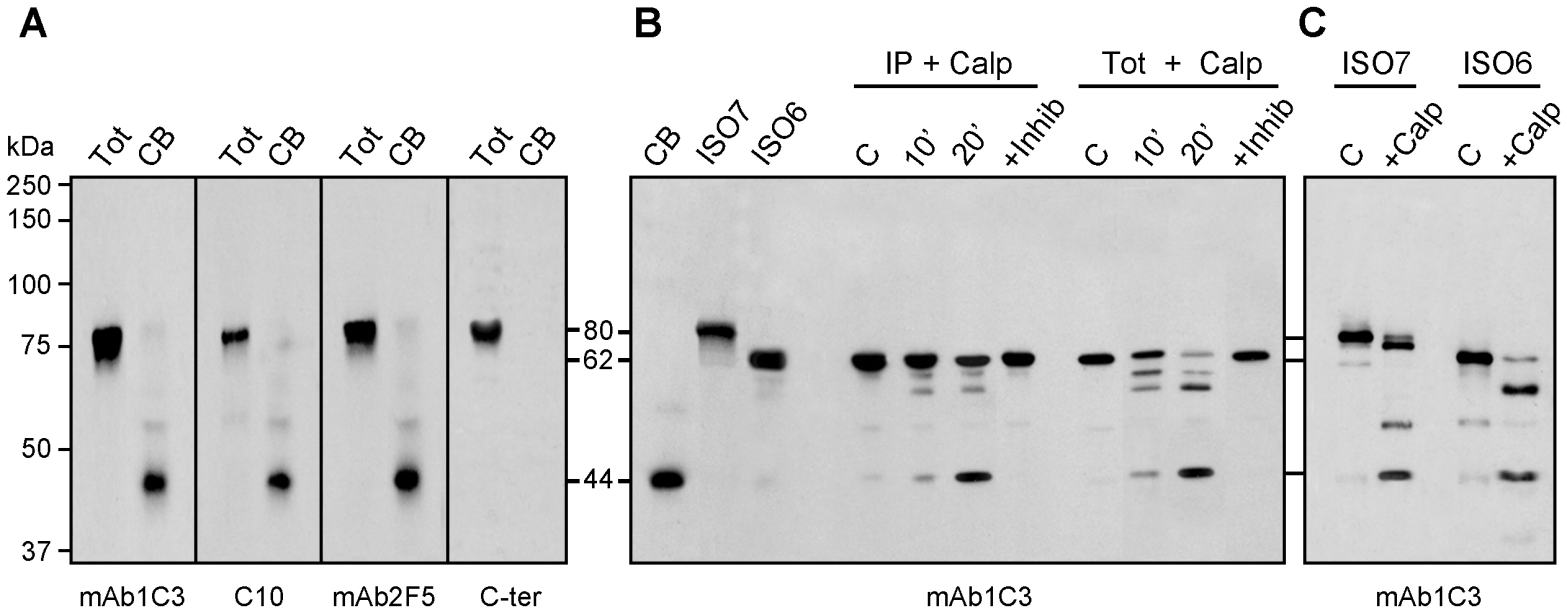
ISO6 FMRP is cleaved by calpain in isolated Cajal bodies. (A) The detected FMRP associated with the Cajal bodies has an apparent molecular weight lower than expected for ISO6 FMRP. Immunoblot analyses of FMRP present in Cajal bodies using different antibodies to FMRP. (B) ISO6 FMRP is a substrate for calpain1. Assays were carried out either with immunoprecipitated ISO6 FMRP or with total cell lysate in the presence of 0.05 U of Calpain 1 for 10 and 20 min at room temperature. The reaction was inhibited in the presence of ALLN (+ Inhib). C : control reaction without the enzyme. (C) Comparison of cleavage products and intermediates between ISO6 and ISO7 FMRP. FMRP species were revealed with four different antibodies in (A) and with mAb1C3 in (B,C).

To demonstrate biochemically the presence of ISO6 and ISO12 FMRP in Cajal bodies, we isolated and purified these structures according to the procedure described by the Lamond's laboratory [38], [39]. Immunoblot analyses of Cajal body proteins using the FMRP mAb1C3 revealed a band at approximately 44 kDa (Figure 7A). The same band was observed using mAb2F5 directed against an epitope laying between amino acids 1 and 204 of FMRP [40], as well as with IgYC10. All three of these antibodies react with ISO1, 6, 7 and 12. In contrast, a rabbit polyclonal antibody directed against the FMRP peptide RTGKDRNQKKEKPD (amino acids 557 to 619) present at the C-terminus of full-length FMRP (ISO1) did not react with the Cajal extracts. Since this peptide sequence is present in ISO1 and 7, but not in ISO6 and 12, due to the frameshift induced by alternative splicing of exon 14, these results indicate that only ISO6/12 forms of FMRP associate with Cajal bodies. The unexpected observation that reactive FMRP in isolated Cajal bodies migrates at 44 kDa strongly suggests that ISO6/12 nuclear proteins are processed. Such a processing has been recently described for two well-known Cajal bodies markers, namely SMN and Coilin, which have been shown to be targets of calpain [41]–[43]. Contrary to proteases that fully degrade a substrate protein, calpains are calcium-dependent cysteine proteases that act by limited specific cleavages. We therefore examined whether the 44 kDa FMRP reactive protein could correspond to ISO6/12 that had undergone limited cleavage of the full length proteins, which have apparent molecular weights of 62 and 60 kDa respectively (see Figure S3). Bioinformatic searches [44] predict that the highest scoring calpain cleavage site is situated at amino acid 369, yielding an FMRP form with a theoretical molecular weight of 42 kDa, a value close to the observed apparent molecular weight of 44 kDa obtained in SDS-PAGE.

To determine if the 44 kDa product we observed is generated by Calpain digestion, we assayed the cleavage susceptibility of ISO6 in a cell-free assay. This nuclear isoform was used as a model since it is the longest nuclear isoform. ISO6 was transiently expressed in STEK *Fmr1* ^−/−^ KO cells that lack FMRP, and cell lysates were incubated in the presence of IgYC10 and anti-chicken antibodies coupled to agarose beads. The immunocomplex was treated *in situ* in the presence of 0.05 U Calpain1 for 10 and 20 minutes. As controls, the complexes were either not treated, or treated in the presence of ALLN, an inhibitor of Calpain 1. The results (Figure 7B) clearly showed that a FMRP fragment at approximately 44 kDa was generated after incubation of ISO6 with Calpain1. Additional bands were detected around 60 and 57 kDa, that we interpreted to be intermediate cleavage products. Since the majority of FMRP present in the immunocomplex attached to the agarose beads yielded only partial cleavage, we hypothesized that the cleavage sites could be structurally protected in the immunocomplex preventing its complete digestion. We therefore conducted the Calpain 1 assay using total extracts obtained from STEK cells transfected with pTL1-ISO6 *FMR1*. A clearer picture was obtained since a progressive decrease of ISO6 could be followed while intermediate species were generated (Figure 7B). Finally, we compared the digestion patterns of ISO6 and ISO7, which showed the main cleavage product at 44 kDa, while intermediate cleavage products (Figure 7C) were different in agreement with the fact that ISO7 contains additional cleavage sites.

### FMRP ISO6 from Cajal bodies binds homopolymer RNA

**Figure 8:**
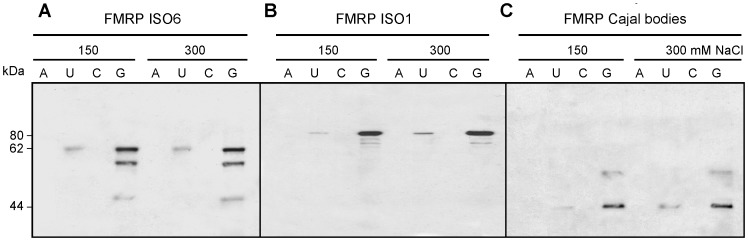
ISO6 binds to RNA homopolymers. Extracts from STEK *Fmr1* ^−/−^ KO cells expressing ISO6 (A) and ISO7 (B) were mixed with agarose beads carrying polyA, polyU, polyC and polyG homopolymers. Captured proteins in the presence of 150 and 300 mM NaCl were eluted with SDS-sample buffer and analyzed by immunoblotting using mAb1C3. (C) Cleaved ISO6 in Cajal bodies also binds preferentially to polyG and to a lesser extend to polyU as is the case for ISO7 and ISO1 (not shown) FMRP.

In the absence of any information about putative RNAs that would bind ISO6, we performed RNA binding assays using homopolymer RNAs conjugated to agarose beads [45] to determine the ability of ISO6 to bind RNA compared to its full-length ISO1 counterpart. Despite the fact that it lacks the C-terminal RGG domain present in ISO1, we observed that ISO6 was preferentially retained on polyG and to a lesser extent to polyU, but not to polyA or polyC (Figure 8B), a pattern of binding to RNA homopolymers similar to that observed for FMRP ISO1 [5]. Since our studies suggested that ISO6 is processed in Cajal bodies to yield a 44 kDa protein lacking the C-terminus, but still retaining the KH1 and KH2 domains, we examined the RNA binding of the cleaved ISO6 from extracted Cajal bodies. We observed the same binding patterns seen with full length ISO6. The binding activity to polyG and polyU was still stable at 300 mM NaCl. These results strongly suggest that the affinity of ISO6 to polyG is not due to the RGG domain, but rather to the intrinsic properties of the KH1 and KH2 domains, that are shared by the different FMRP isoforms. Our results suggest that ISO6 might also play a role in interacting with RNA in Cajal bodies.

### FMRP ISO6-I304N is defective in Cajal body association

**Figure 9:**
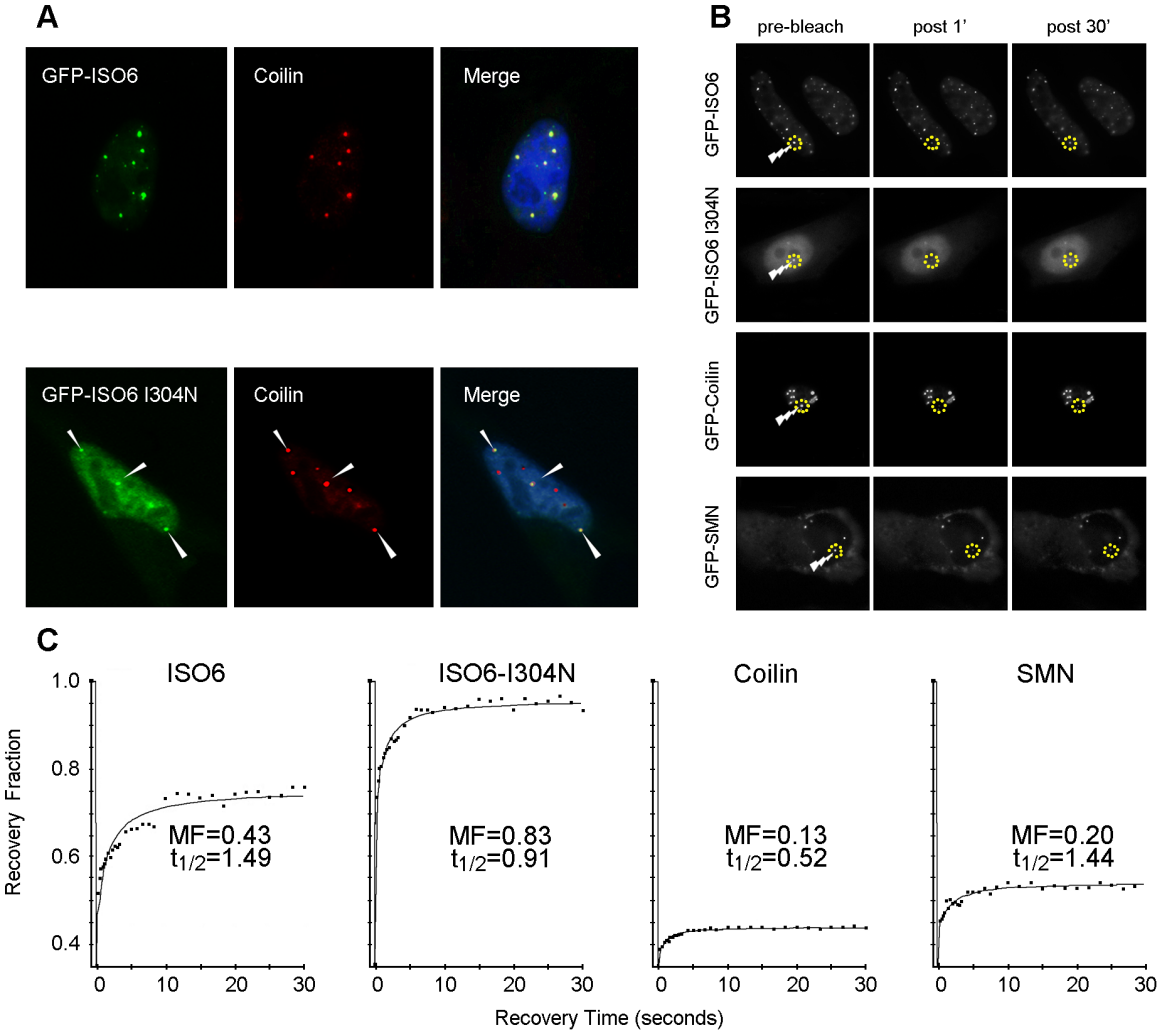
ISO6-I304N FMRP is defective in its Cajal body association. (A) GFP-ISO6-I304N displays reduced association with Cajal bodies. Note that not all coilin positive structures contain ISO6-I304N and that the majority of the ISO6-I304N remains nucleoplasmic. Arrowhead point to mini-ISO6-I304N positive Cajal bodies. (B) HeLa cells transiently transfected with the indicated GFP-fusion construct were used for FRAP experiments (as described in Material and Methods). (C) GFP-ISO6-I304N displays a faster turnover rate relative to wild type FMRP-ISO6.

Our homopolymer binding assays indicated that the RNA binding properties of the KH domains of processed FMRP ISO6 were conserved. It was previously shown that a missense mutation of an isoleucine to asparagine (I304N) in the second KH-type RNA-binding domain, identified in a patient with a severe Fragile X phenotype [46], greatly affects the RNA binding properties of FMRP [47]–[49]. We therefore introduced this mutation in the GFP-FMRP-ISO6 construct in order to study the nuclear fate of the mutant protein. In repeated experiments, we consistently observed that ISO6-I304N displayed a significantly diminished association with Cajal bodies, and was found mostly in the nucleoplasm (Figure 9A). These results strongly suggest that the RNA-binding properties of FMRP-ISO6 are necessary for the protein to be incorporated in Cajal bodies.

We next reasoned that this reduced or partial co-localization of ISO6-I304N with Cajal bodies might be a reflection of differential dynamic behavior. In order to study the dynamic turnover of ISO6 and ISO6-I304N in Cajal bodies, we performed Fluorescence Recovery After Photobleaching (FRAP) experiments (Figure 9B). Indeed, the kinetics of recovery of a photobleached GFP-tagged protein can be seen as a reflection of its degree of association with other proteins and/or nucleic acids. For these experiments, HeLa cells were transfected with either GFP-ISO6, GFP-ISO6-I304N, GFP-coilin or GFP-SMN for comparison purposes, and cells were imaged after ∼6 h post-transfection as above. Briefly, one fluorescent protein-containing Cajal body per cell nucleus was photobleached with a brief laser pulse and a series of images then captured in rapid succession (Figure 9B). Subsequent quantification of the fluorescent intensities within the photobleached area plotted over time was used to derive mobile fraction (MF) and half time of recovery (t_1/2_) values. Using this approach, we obtained a MF of 0.43 with a t_1/2_ of 1.5 sec for GFP-FMRP-ISO6 in Cajal bodies (Figure 9C). This represents significantly faster kinetics than the other Cajal body-resident proteins GFP-coilin and GFP-SMN (Figure 9C), which both display comparable and slower dynamic turnover as documented previously [31], [50]. Strikingly, a much faster turnover rate was obtained for GFP-ISO6-I304N mutant (Figure 9C), consistent with this mutation likely disrupting one or more binding events as predicted. Altogether, these findings provide evidence for a novel function of the FMRP isoforms ISO6 and/or ISO12 in the nucleus, likely involving interaction with RNA. Most importantly, our results with the I304N patient mutation also suggest that loss of this novel nuclear function might contribute to the Fragile X syndrome. 

## Discussion

**Figure 10:**
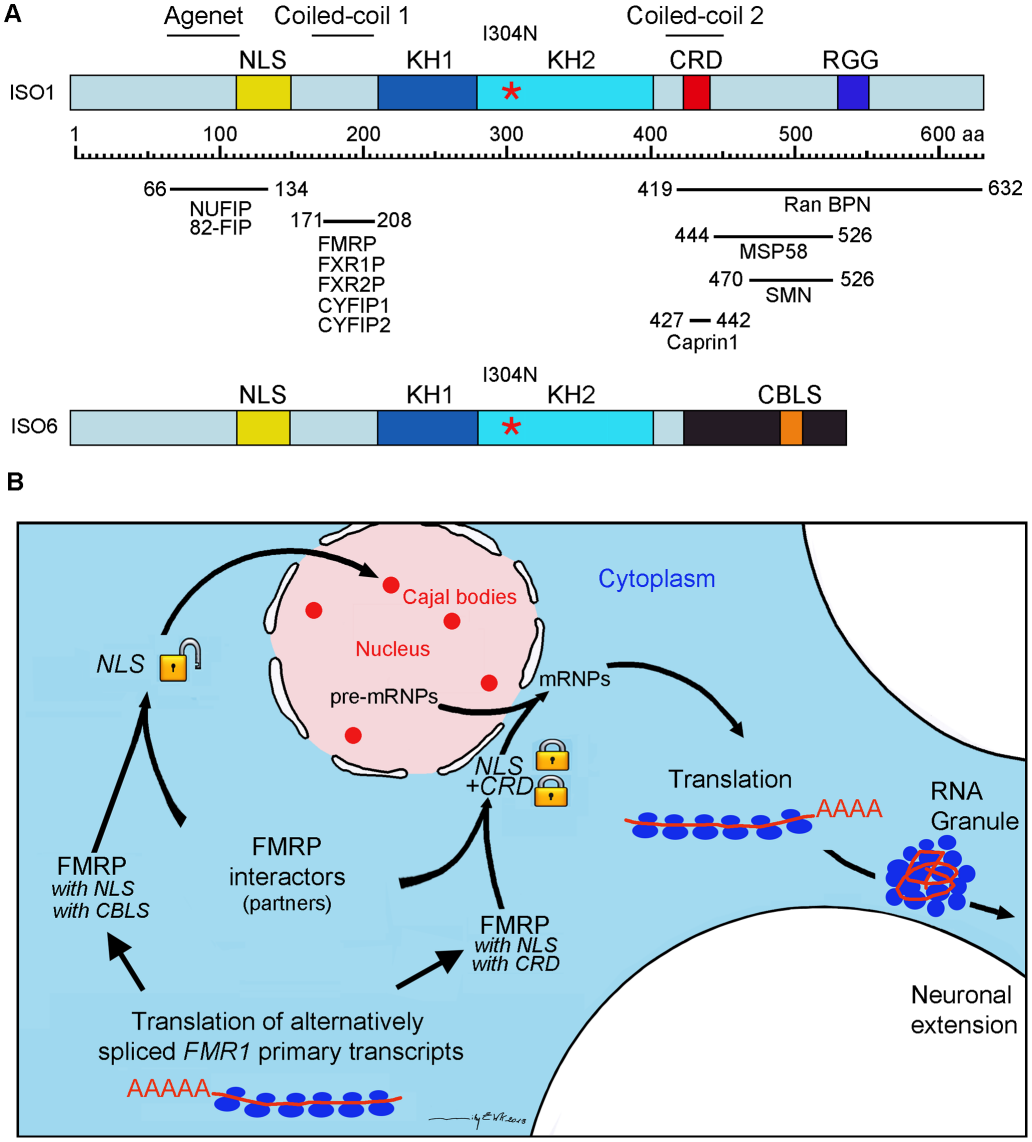
Proposed model for the fate of the nuclear ISO6 and full length ISO1 FMRP. (A) Schematic representation of ISO1 and ISO6 FMRP and mapping of known protein partners. Note the position of the Cajal Body Localization Signal (CBLS). (B) Alternative splicing of the primary transcripts generates either ISO6 FMRP lacking the CRD domain, or ISO1 FMRP containing both NLS and CRD domains. ISO6 is driven to Cajal bodies by transporter proteins, while ISO1 interacts with protein partners that lock the NLS and CRD domains and is localized to the perinuclear area to join the nascent mRNPs complexes emerging from the nuclear pores. In the cytoplasm the ISO1 FMRP-mRNPs particles associate with the translation machinery or are transported in RNA-granules to micro-domains away from the soma.

FMRP is a cytoplasmic protein associated with the translation apparatus. However, it has been initially reported that FMRP was observed as intense nuclear staining in esophageal epithelium of mice [16], but the reasons for this singular presence in nuclei remain obscure. Despite 20 years of intense efforts to detect FMRP in the nucleus in cell cultures, even when using the power of transfection assays, it is puzzling that the highest score ever reported is 0.4% of nuclei positive for FMRP [51]. It is worth mentioning that this estimate was obtained after 38 h of transfection, at a time when cells are overloaded with FMRP, which induces the formation of stress granules containing FMRP (Figure S3) and the repression of translation with all its deleterious consequences [29]. Based on the assumption that FMRP is present in the nucleus, and that it contains NLS and putative NES signals, it has been widely accepted that FMRP is therefore a nucleocytoplasmic shuttling protein. Supporting this model, FMRP has been detected by immunogold electron microscopy in the nucleoplasm and in nuclear pores within neurons of rat brain, and it was deduced from the obtained images that FMRP was in transit between the nucleus and the cytoplasm [15]. However, it is important to recall that the mAb1C3 used (previously also referred as to mAb1a) is directed against an epitope laying between amino acids 66 and 112 at the N-terminus of FMRP [4]. Since the N-terminus is maintained and is common to all FMRP isoforms, it is not possible to determine which of the nuclear or cytoplasmic FMRP isoform(s) has(have) been detected. While truncated FMRP lacking the carboxyl portion and the NES, localizes to the nucleus [4], the intact protein with the NES is cytoplasmic and barely penetrates the nucleus even when fused to the SV40 large T-antigen NLS [51] if one is careful not to observe FMRP at late times when cells are overly saturated with expressed protein. Indeed, we have observed that when overexpressed in transient transfection assays, FMRP accumulates to such high levels in the cell that any unusual images of distorted FMRP can be interpreted to the taste of the observer (Figure S3). Based on our results, we speculate that the sequence (429-LRLERLQI-438) present in the full length FMRP, although reminiscent of Rev-Rex-PKI NES nuclear export sequences (LPPLERLTL) behaves rather as a cytoplasmic anchoring domain or as a cytoplasmic retention domain (CRD) as proposed earlier [17], [52]. What would then be the factors controlling the activities of the NLS and CRD? FMRP contains two regions predicted to have a significant propensity to form coiled coil motifs involved in protein-protein interactions [52]. As illustrated in Figure 10A, the first coiled-coil domain is situated adjacent to the N-terminal NLS, while the second domain overlaps the C-terminal CRD domain. A third domain, the Agenet, also referred to as the NDF (N-terminal domain of FMRP) [53], also overlaps with the NLS. Interestingly, all three regions are platforms for different known protein-protein interactors as illustrated in Figure 10A. Details of FMRP domains involved in interaction with protein partners can be found in [18], [52]–[59]. By binding to these domains of FMRP, protein partners would lock either the NLS or the CRD domains or both, the result of which will be the retention of FMRP in the cytoplasmic compartment. In agreement with this mechanism, we have recently observed that Caprin1 interacts with FMRP at position 427–442 within the CRD [18]. On the other hand, we propose that isoforms of FMRP lacking the CRD, such as ISO6 and ISO12 have their NLS available to cellular factors that might guide them to the nucleus. Since the C-terminus of ISO6 differs from that of ISO1 (Figure 10A) it is not expected *a priori* that protein partners interacting with ISO1 at its C-terminus would also interact physically with ISO6. On the other hand, as the N-terminus is maintained it is expected that protein partners that associate with ISO1, also interact with ISO6. It is fascinating that NUFIP has been originally shown to interact with ISO12 [59], to shuttle between the nucleus and the cytoplasm [60] and to be implicated in nuclear RNPs biogenesis [61].

Since, according to our model, FMRP full length would not penetrate the nucleus, we postulate that it interacts at the periphery of nuclei with the nuclear pre-mRNPs that are just emerging from the nuclear pores to chaperon them to the translation machinery (Figure 10B). Consistent with this view, cytoplasmic FMRP is concentrated at the periphery of the nucleus, lying in the perinuclear area (see Figure 2B).

Of the four nuclear FMRP isoforms that have been detected and characterized [17], we found that only ISO6 and ISO12 are targeted to Cajal bodies. While the alternately spliced ISO6 and 12 mRNA variants have been shown to be actively translated [35] this is not the case for ISO4 and 10 mRNAs. Bioinformatic analyses predict that the latter two are substrates for the nonsense-mediated mRNA (NMD) pathway.

Previous studies have shown that transiently expressed ISO12 FMRP is predominantly localized in the perinucleolar region [53]. It is worth mentioning that Cajal bodies were originally termed as nucleolar «accessory bodies» as they were detected close or in direct contact with the nucleolus (drawings from Santiago Ramón y Cajal can be found in ref [62]). It is therefore possible that the nucleolar localization of ISO12, shown in [53], represents a static snapshot of dynamic events taking place between Cajal bodies and nucleoli, a phenomenon which has been documented *in vivo* by time lapse microscopy [63].

The fact that ISO6/12 are RNA-binding proteins favors the hypothesis that they could be implicated in nuclear post-transcriptional RNA control. As highlighted by the I304N mutation, the RNA-binding properties of ISO6 FMRP are necessary to allow its localization and perhaps its stability into Cajal bodies. However this hypothesis does not rule out that they might have additional or other function(s) as Cajal bodies have been reported to be involved in histone transcription and 3′-end processing, in assembly and maturation of RNP complexes, including splicing snRNPs, snoRNPs, scaRNPs and the telomerase RNP [reviewed in 20]–[23], [64].

The functional significance of the processing of nuclear FMRP by Calpain 1 remains unknown, as both processed and unprocessed nuclear isoforms are able to bind homopolymer RNAs. It is worth noting that calpain cleavage at amino acid 369 is in the variable loop of the KH2 domain and leaves the 42/44 kDa truncated ISO6/12 with the ancestral KH core domain structure likely to be functional. Processing by calpain has been reported for coilin and SMN [41]–[43], known proteins associated with Cajal bodies. This mechanism might be necessary for their assembly. Further studies using antibodies to different portions of ISO6 FMRP will be required to determine where the processing of ISO6 takes place. A probable location will be in the nucleus, since cytoplasmic FMRP associated with polyribosomes seems to be protected as it is not processed.

The fragile X syndrome by its peculiar mode of inheritance and its unusual dynamic mutations makes exception in the context of classical genetics. The analyses of the functions of the numerous isoforms as well as of their differential and complex expression pattern in different tissues [35] may hold further surprises. We believe that the present study opens unexplored avenues in search for new insights into the pathophysiology of Fragile X syndrome.

## Materials and Methods

### Cell cultures

HeLa S_3_ cell line was purchased from ATCC, and STEK *Fmr1* ^−/−^ KO cell line was established as previously described [29]. Motoneuron-derived MN-1 cells have been described previously [65]. Cultures from human fibroblasts were obtained from Mahmoud Rouabhia (Faculté de médecine dentaire, Université Laval, Québec, Canada). Fragile X fibroblasts GM05131 and GM05848 were obtained from Coriell Cell Repository (Camden, NJ, USA). Cells were propagated and maintained in DMEM supplemented with 10% FBS and antibiotics (100 units/ml penicillin, 50 mg/ml streptomycin). For transfection assays, Lipofectamine 2000 (Invitrogen) was used according to the manufacturer's protocol. Treatments with Leptomycin B (Sigma) were performed at the indicated doses and periods as detailed in the Results section.

### Protein studies

#### Antibodies

IgY from egg yolks were purified using the Eggcellent chicken IgY purification kit from Pierce. IgY specific for FMRP were affinity purified from total IgY as described [18]. FMRP was detected using hybridoma supernatants from mAb1C3, previously referred as to mAb1a [4], mAb2F5 [40], (obtained from the Developmental Studies Hybridoma Bank, University of Iowa, USA), and affinity purified rabbit IgG directed against FMRP C-terminus (OAEB012228 Aviva Sys Bio). Coilin was detected with affinity purified rabbit anti Coilin (Protein Tech), SMN with mAb8/SMN (BD Transduction Lab), and β-tubulin with mAbE7 (obtained from the Developmental Studies Hybridoma Bank, University of Iowa, USA).

#### Immunoblot analyses

Protein separated on SDS-PAGE (8% acrylamide) were transferred onto 0.45 µm nitrocellulose (BioRad) and processed for immunodetection after blocking in 5% nonfat dry milk in PBS. The dilutions for the primary antibodies were : mAb1C3 and mAb2F5 hybridoma supernatants 1∶1; IgYC10, 1∶5000; OAEB01228, 1∶1000. Detection of bound antibodies was performed with HRP-coupled goat secondary antibodies to mouse, chicken or rabbit (Immunoresearch) followed by ECL reaction (Perkin Elmer).

#### Calpain assay

pTL1-ISO6 and ISO1 *FMR1* were expressed in STEK *Fmr1* ^−/−^ KO cell line. Cells were lysed in a buffer made of 50 mM Tris-HCl, pH 7.6, 150 mM NaCl, 2.5 mM MgCl_2_ and 1% NP-40 supplemented with Protease Inhibitor Cocktail (Roche). Assays were carried out either with cell lysates or with immunoprecipitated FMRP using IgYC10 as described [18] in the presence of 0.05 U of Calpain 1 (Calbiochem) and 1 mM CaCl_2_ and incubated at room temperature for 10 and 20 minutes. To inhibit calpain activity, 0.5 mM N-acetylleucylleucylnorleucinal (ALLN; Sigma Aldrich) was added to the respective reactions. The reaction was stopped by addition of SDS-sample buffer prior to immunoblot analyses.

#### Immunofluorescence analyses

Cells grown on coverslips were rinsed twice with cold PBS and fixed in the presence of 4% para-formaldehyde in PBS for 10 minutes at room temperature followed by permeabilization with 0.25% Triton X-100 in PBS for 10 minutes at room temperature. Primary antibodies were diluted in PBS containing 3% BSA. The incubations with the first and second antibodies were at room temperature for 60 minutes for each. Secondary antibodies were: goat anti-mouse Alexa 488, goat anti-rabbit 488 and 546, and goat anti-chicken Alexa 546 (Invitrogen). Coverslips were mounted in Prolong Gold Antifade mounting media (Invitrogen). Images were acquired using a Zeiss LSM510 confocal microscope and treated using the *MetaMorph* software.

#### Live cell imaging

FRAP experiments were carried out essentially as described [66] using a wide-field fluorescence microscope (DeltaVision CORE; Applied Precision, Issaquah, WA, USA) equipped with a three-dimensional motorized stage, temperature- and gas-controlled environmental chamber, and 488-nm diode laser (for photobleaching EGFP). Images were collected using a 60X NA 1.4 Plan-Apochromat objective and recorded with a CoolSNAP coupled-charge device (CCD) camera (Roper Scientific, Trenton, NJ, USA). The microscope was controlled by SoftWorX acquisition and deconvolution software (Applied Precision). For FRAP experiments, a single section was imaged before photobleaching, a region of interest was then bleached using the 488-nm laser, and a rapid series of images was acquired after the photobleaching period. Recovery curves were plotted and the mobile fraction and half time of recovery were determined using SoftWorX.

### Nucleic acids studies

#### cDNA constructs

pTL1 expression vectors carrying ISO1, ISO4, ISO6, ISO7, ISO10 and ISO12 *hFMRP* cDNAs were described in Sittler *et al.* [17]. The corresponding cDNAs inserts were excised from the pTL1 plasmids using EcoR1-Pst1 digestion, and the resulting fragments subcloned into the pmeGFP-C2 plasmid carrying an A207K mutation in the GFP to eliminate GFP-dependent aggregation [67]. pBS vectors carrying mouse *Fmr1* Iso6 (obtained from David Morris, University of Washington, Seattle, USA [35]) was digested by ApaI/SalI, and the resulting cDNA fragment subcloned into the pmeGFP-C1 vector to generate pmeGFP-m*Fmr1* Iso6. To generate deletion mutant meGFP-*hFMR1* ISO6Δ489-505, pmeGFP-*hFMR1* ISO6 plasmid was used as template for PCR-mediated plasmid DNA deletion method. Primers were designed to amplify the entire sequence of the plasmid except for the specific region that was to be deleted. The sequences of the primers synthesized were: 5′-ATGGATCCCTTCAGAATACCTCCAGTGAAGGTAG-3′ and 5′-CTTCACTGGAGGTATTCTGAAGGGATCCATCTGT-3′.

#### Other constructs

GFP-coilin: the human cDNA for p80-coilin was obtained using RT-PCR from HeLa cells total RNA and inserted into the pEGFP-C1 vector at Kpn1 and BamH1 sites. GFP-SMN: the human cDNA for SMN was obtained using RT-PCR from HeLa cells total RNA and inserted into the pEGFP-C1 vector at EcoR1 and BamH1 sites. All constructs were verified by sequencing.

#### Homopolymers binding

Binding assays were performed according to established procedures [45]. Briefly, 0.3 mg of total cytoplasmic protein extracted from STEK *fmr1* ^−/−^ KO cell line transfected with either pTL1-ISO6 or pTL1-ISO7 were incubated with immobilized polyG, polyU, polyA or polyC polyacrylhydrazido-agarose beads (Sigma) in 0.5 ml of binding buffer containing 10 mM Tris–HCl, pH 7.4, 150 or 300 mM NaCl, 2.5 mM MgCl_2_, 0.5% Triton X-100 supplemented with Protease Inhibitor Cocktail for 10 min on a rocking platform at 4°C. After incubation, the beads were pelleted after a brief spin in a microfuge and washed four times with binding buffer and bound proteins eluted by addition of SDS sample buffer followed by heat denaturation. Proteins were separated by SDS–PAGE and immunoblotted using mAb1C3 followed by ECL reaction.

### Sub-cellular fractionation

#### Cell fractionation

HeLa cells grown in petri dishes were washed twice with cold PBS and lyzed in the presence of 20 mM Tris-HCl, pH 8.0, 150 mM NaCl, 1.25 mM MgCl_2_, 1% NP40, supplemented with Protease Inhibitor Cocktail. The lysates were homogenized by passage through a 1 ml syringe mounted with 18, 23 and 28 gauge needles, successively. The homogenates were centrifuged at 2 000 rpm for 5 minutes to yield an enriched nuclear fraction and a cytoplasmic supernatant. The nuclear fraction was washed once and the supernatants pooled. All manipulations were performed at 4°C. Aliquots from total, cytoplasmic and nuclear fractions were heat denaturated in 3× concentrated SDS-sample buffer and analyzed by immunoblotting to detect FMRP.

#### In situ cell fractionation

HeLa cells grown on coverslips in 35 mm diameter petri dishes were rinsed twice with cold PBS and lyzed *in situ* in the presence of a buffer containing 20 mM Tris-HCl, pH 7.4, 150 mM NaCl, 1.25 mM MgCl_2_, 1% NP40, supplemented with Protease Inhibitor Cocktail and 1 mM DTT, 50 µg/ml cycloheximide, and 5 U RNasin (New England Biolabs). The petri dish containing the coverslip was gently swirled at 4°C for 10 minutes on an orbital shaker at low speed, and the supernatant was discarded. After washing twice with PBS, the remaining material attached to the coverslips was fixed with 4% paraformaldehyde in PBS for 5 minutes at room temperature, washed twice with PBS and processed for immunofluorescence.

#### Cajal bodies preparation

Eight to ten ×10^8^ HeLa cells were used for each single preparation of Cajal bodies following the detailed protocols of Lamond's laboratory [38], [39].

## Supporting Information

Figure S1 FMRP is detected as perinuclear granules with mAb1C3 after gentle lysis of HeLa cells.
(TIF)

Figure S2 Immunoblot analysis of transiently expressed FMRP isoforms. Whole cell extracts (10 µg) of STEK *Fmrp1* ^−/−^ KO cells transfected with ISO1, ISO7, ISO6, ISO12, ISO4 and ISO10 pTL1 expression vectors were separated by SDS-PAGE (8% acrylamide) and revealed with IgYC10 followed by ECL.
(TIF)

Figure S3 Cellular localization of transfected FMRP isoforms. pTL1 expression vectors coding for ISO1 (A), ISO7 (B) and ISO6 (C) FMRP were transfected in Cos cells and analyzed at 38 h post-transfection. Note the intense cytoplasmic fluorescence in stress granules surrounding the nucleus in (A) and (B), while fluorescence is excluded from the nuclei. In (C) is shown the perinucleolar localization of ISO6 as ring-shaped structures. FMRP was revealed with mAb1C3 followed by anti-mouse Ig secondary antibodies.
(TIF)

Figure S4 Exon structure of the human *FMR1* gene generating different isoforms through utilization of both differential splice-sites and alternate reading frames in exon 15–17. (A) exon 15; (B) exon 16; and (C) exon 17. Alternate transcription of the human *FMR1* gene generates isoforms either lacking or containing exon 14. Isoform 1 (ISO1) utilizes exon 14 which is spliced into exon 15 using the proximal splice acceptor (SA) and generates a protein sequence encoded by reading frame 3 (RF3) through exon 15a (grey) and exon 15 b (blue) (A) and reading frame 3 through exon 16 (blue). (B) In Iso1, exon 16 is spliced into exon 17 using the proximal SA and generates the C-terminal protein sequence in reading frame 1 (RF1) through exon 17a (grey) and exon 17b (blue) (C). Isoform 6 (ISO6) does not utilize exon 14 and exon 13 is spliced into exon 15 using the distal SA site and generates a protein encoded by RF1 through exon 15b (yellow), and RF1 through exon 16 (yellow). In ISO6, exon 16 is spliced into exon 17 using the proximal SA and generates the C-terminal protein sequence using RF2 through exon 17a (grey) and exon 17b (yellow). Numbering is from the human FMR1 gene – Accession L29074; Exons labeled «a» derive from the proximal SA (coding sequences are highlighted in gray) and those labeled «b» derive from the distal SA. RF = reading frame, determined from the exon sequence of the proximal SA, ie RF1 = codons beginning at bp1 of the exon, etc.
(DOC)

Figure S5 Conservation of proximal and distal splice acceptor sites in exon 17 of human and mouse FMR1. The genomic DNA sequence of the human and mouse FMR1 gene are shown within exon 17. The highly conserved proximal and distal splice acceptor (SA) sites within exon 17 are highlighted in red. The protein sequences encoded in different reading frames for both mouse and human FMR1 isoforms are shown with sequences encoded by exon 17a from the proximal SA (grey) and sequences encoded by exon 17b from the distal SA: ISO1 (blue) and ISO6 (yellow). pred = computer prediction from genomic sequence; SA = splice acceptor site; var = isoform variant. The references for the experimentally determined cDNA and EST clones are indicated. ^a^cDNA clone [17]; ^b^EST clones: HY131001, CX756143; ^c^cDNA clone [33], S65791; ^d^EST clone : BU554239; ^e^cDNA clone *mIso6* [32], [35].
(DOC)
