# Supplementary figures and images for: Nuclear Fragile X Mental Retardation Protein Is localized to Cajal Bodies
Source: PLoS Genet. 2013 Oct 31;9(10):e1003890. doi: 10.1371/journal.pgen.1003890 (PMC3814324; doi:10.1371/journal.pgen.1003890)

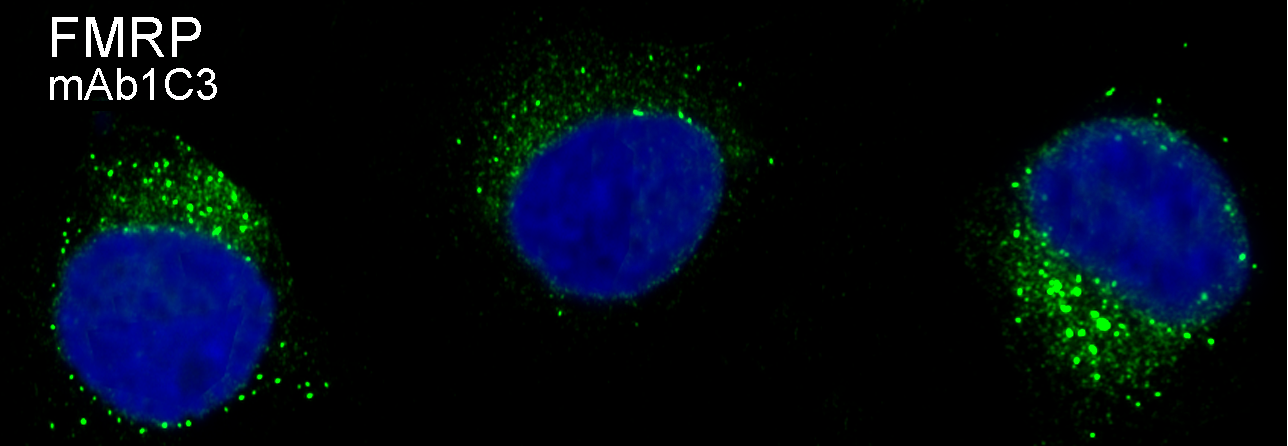

Supplement: Figure S1 — FMRP is detected as perinuclear granules with mAb1C3 after gentle lysis of HeLa cells. (TIF) [file pgen.1003890.s001.tif]

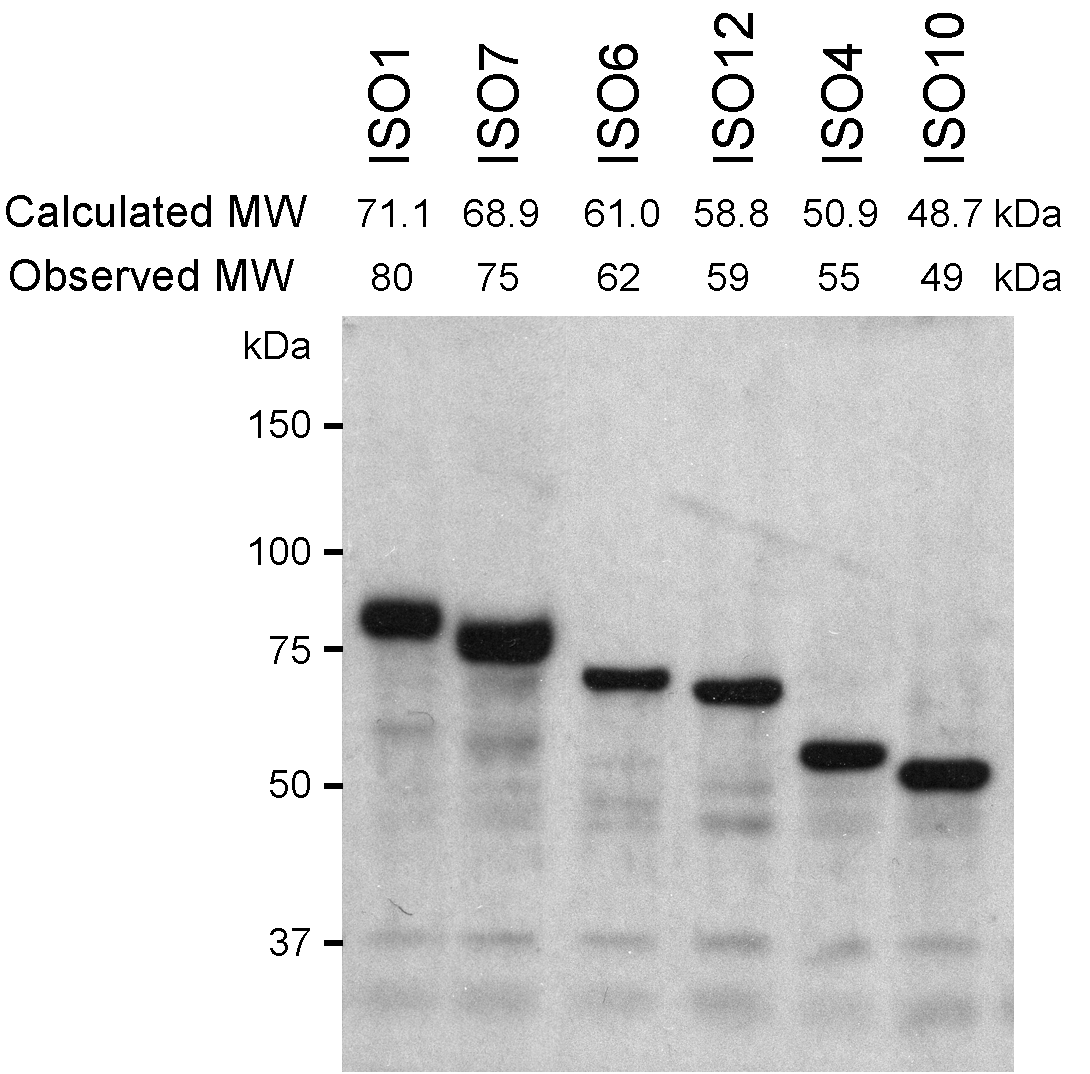

Supplement: Figure S2 — Immunoblot analysis of transiently expressed FMRP isoforms. Whole cell extracts (10 µg) of STEK Fmrp1 −/− KO cells transfected with ISO1, ISO7, ISO6, ISO12, ISO4 and ISO10 pTL1 expression vectors were separated by SDS-PAGE (8% acrylamide) and revealed with IgYC10 followed by ECL. (TIF) [file pgen.1003890.s002.tif]

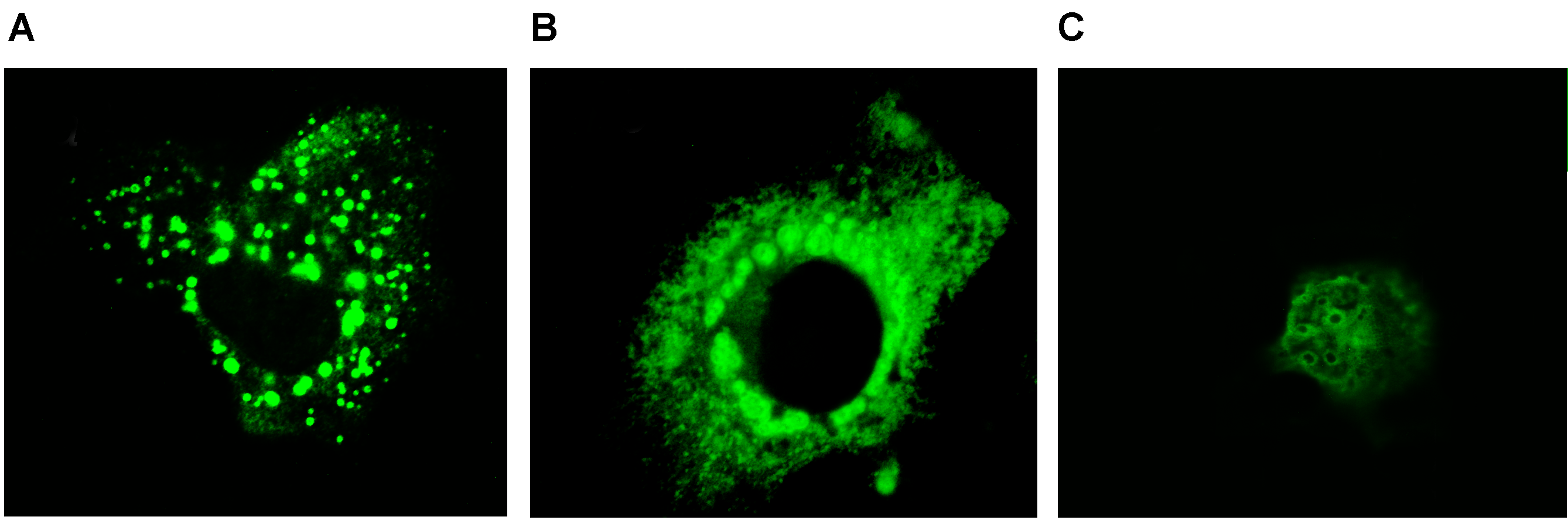

Supplement: Figure S3 — Cellular localization of transfected FMRP isoforms. pTL1 expression vectors coding for ISO1 (A), ISO7 (B) and ISO6 (C) FMRP were transfected in Cos cells and analyzed at 38 h post-transfection. Note the intense cytoplasmic fluorescence in stress granules surrounding the nucleus in (A) and (B), while fluorescence is excluded from the nuclei. In (C) is shown the perinucleolar localization of ISO6 as ring-shaped structures. FMRP was revealed with mAb1C3 followed by anti-mouse Ig secondary antibodies. (TIF) [file pgen.1003890.s003.tif]
