# Supplementary material for: Nuclear Fragile X Mental Retardation Protein Is localized to Cajal Bodies
Source: PLoS Genet. 2013 Oct 31;9(10):e1003890. doi: 10.1371/journal.pgen.1003890 (PMC3814324; doi:10.1371/journal.pgen.1003890)
Supplement: Figure S4 — Exon structure of the human FMR1 gene generating different isoforms through utilization of both differential splice-sites and alternate reading frames in exon 15–17. (A) exon 15; (B) exon 16; and (C) exon 17. Alternate transcription of the human FMR1 gene generates isoforms either lacking or containing exon 14. Isoform 1 (ISO1) utilizes exon 14 which is spliced into exon 15 using the proximal splice acceptor (SA) and generates a protein sequence encoded by reading frame 3 (RF3) through exon 15a (grey) and exon 15 b (blue) (A) and reading frame 3 through exon 16 (blue). (B) In Iso1, exon 16 is spliced into exon 17 using the proximal SA and generates the C-terminal protein sequence in reading frame 1 (RF1) through exon 17a (grey) and exon 17b (blue) (C). Isoform 6 (ISO6) does not utilize exon 14 and exon 13 is spliced into exon 15 using the distal SA site and generates a protein encoded by RF1 through exon 15b (yellow), and RF1 through exon 16 (yellow). In ISO6, exon 16 is spliced into exon 17 using the proximal SA and generates the C-terminal protein sequence using RF2 through exon 17a (grey) and exon 17b (yellow). Numbering is from the human FMR1 gene – Accession L29074; Exons labeled «a» derive from the proximal SA (coding sequences are highlighted in gray) and those labeled «b» derive from the distal SA. RF = reading frame, determined from the exon sequence of the proximal SA, ie RF1 = codons beginning at bp1 of the exon, etc. (DOC) [file pgen.1003890.s004.doc]

A

T N S E A S N A S RF3

46621 gacaatggtatataa**cttttaactctcgatag**gaactaattctgaagcatcaaatgcttc

***Proximal SA***

**Exon 15a Exon 15b**

E T E S D H R D E L S D W S L A P T E E RF3

* L Q Q R K RF1

46681 tgaaacagaatctgaccacagagacgaa**ctcagtgattggtcattag**ctccaacagagga

***Distal SA***

E R E S F L R R G D G R R R G G G G R G RF3

R G R A S C A E E T D G G V E G E E E D RF1

46741 agagagggagagcttcctgcgcagaggagacggacggcggcgtggagggggaggaagagg

Q G G R G R G G G F K G RF3

K E E E D V E E A S K RF1

46801 acaaggaggaagaggacgtggaggaggcttcaa**aggt**atggagatcttcattaagaaatc

***SD***

B **Exon 16**

G N D D H S R T D N R P R N P R E A RF3

E T T I T P E Q I I V H V I Q E R L RF1

47311 **ctcatag**gaaacgacgatcactcccgaacagataatcgtccacgtaatccaagagaggct

***SA***

K G R T T D G S L Q RF3

K E E Q Q M D P F R  RF1

47371 aaaggaagaacaacagatggatcccttc**aggt**aaaacctgtctgcctctttcatcttaat

***SD***

C

50401 tgtcaggccaattacagattacagtaggatatggtctgtgtatataacaa**ctataacttg**

***Proximal SA***

Exon 17a

I R V D C N N E R S V H T K T L Q N RF1

S E L T A I M K G V S T L K H Y R **I** RF2

50461 **ttttag**atcagagttgactgcaataatgaaaggagtgt**ccacactaaaacattacag**aat

**Distal SA**

Exon 17b

T S S E G S R L R T G K D R N Q K K E K RF1

P P V K V V G C A R V K I V T R R K R S RF2

50521 acctccagtgaaggtagtcggctgcgcacgggtaaagatcgtaaccagaagaaagagaag

P D S V D G Q Q P L V N G V P * RF1

Q T A W M V S N H S * RF2

50581 ccagacagcgtggatggtcagcaaccactcgtgaatggagtaccctaaactgcataattc
