# Supplementary material for: Nuclear Fragile X Mental Retardation Protein Is localized to Cajal Bodies
Source: PLoS Genet. 2013 Oct 31;9(10):e1003890. doi: 10.1371/journal.pgen.1003890 (PMC3814324; doi:10.1371/journal.pgen.1003890)
Supplement: Figure S5 — Conservation of proximal and distal splice acceptor sites in exon 17 of human and mouse FMR1. The genomic DNA sequence of the human and mouse FMR1 gene are shown within exon 17. The highly conserved proximal and distal splice acceptor (SA) sites within exon 17 are highlighted in red. The protein sequences encoded in different reading frames for both mouse and human FMR1 isoforms are shown with sequences encoded by exon 17a from the proximal SA (grey) and sequences encoded by exon 17b from the distal SA: ISO1 (blue) and ISO6 (yellow). pred = computer prediction from genomic sequence; SA = splice acceptor site; var = isoform variant. The references for the experimentally determined cDNA and EST clones are indicated. acDNA clone [17]; bEST clones: HY131001, CX756143; ccDNA clone [33], S65791; dEST clone : BU554239; ecDNA clone mIso6 [32], [35]. (DOC) [file pgen.1003890.s005.doc]

**EXON 17a Exon 17b**

**hISO6 (cDNA^a^)>** S E L T A I M K G V S T L K H Y R I P P V *(RF2)*

**hISO6var (pred)>** I P P V *(RF2)*

**hISO1var (EST^b^)>** N T S S *(RF1)*

**hISO1 (cDNA^c^)>** I R V D C N N E R S V H T K T L Q N T S S *(RF1)*

**proximal SA distal SA**

**hFMRP**  TAACTTGTTTTAGATCAGAGTTGACTGCAATAATGAAAGGAGTGTCCACACTAAAACATTACAGAATACCTCCAGTG

||||||||||||||||| |||| ||||||||||||||| || |||||| |||||| ||||||||| | |||||||||

**mFMRP**  TAACTTGTTTTAGATCACAGTTAACTGCAATAATGAAAAGACTGTCCATACTAAACCATTACAGAGTGCCTCCAGTG

**mISO1 (pred)>** I T V N C N N E R T V H T K P L Q S A S S *(RF1)*

**mISO1var (EST^d^)>** S A S S *(RF1)*

**mISO6 (pred)>** S Q L T A I M K R L S I L N H Y R V P P V *(RF2)*

**mISO6var (mIF6;cDNA^e^)>** V P P V *(RF2)*
